# Prevalence of Herpes and Respiratory Viruses in Induced Sputum among Hospitalized Children with Non Typical Bacterial Community-Acquired Pneumonia

**DOI:** 10.1371/journal.pone.0079477

**Published:** 2013-11-18

**Authors:** Weimin Zhou, Feng Lin, Lingfang Teng, Hua Li, Jianyi Hou, Rui Tong, Changhua Zheng, Yongliang Lou, Wenjie Tan

**Affiliations:** 1 Key Laboratory of Medical Virology, Ministry of Health; National Institute for Viral Disease Control and Prevention, China CDC, Beijing, China; 2 Wenling Affiliated Hospital, Wenzhou Medical College, Wenling, Zhejiang, China; 3 Institute of Medical Virology, Wenzhou Medical College, Wenzhou, Zhejiang, China; University of Kansas Medical Center, United States of America

## Abstract

**Objective:**

Few comprehensive studies have searched for viruses in infants and young children with community-acquired pneumonia (CAP) in China. The aim of this study was to investigate the roles of human herpes viruses (HHVs) and other respiratory viruses in CAP not caused by typical bacterial infection and to determine their prevalence and clinical significance.

**Methods:**

Induced sputum (IS) samples were collected from 354 hospitalised patients (infants, n = 205; children, n = 149) with respiratory illness (CAP or non-CAP) admitted to Wenling Hospital of China. We tested for HHVs and respiratory viruses using PCR-based assays. The epidemiological profiles were also analysed.

**Results:**

High rate of virus detection (more than 98%) and co-infection (more than 80%) were found among IS samples from 354 hospitalised infants and children with respiratory illness in this study. Of 273 CAP samples tested, CMV (91.6%), HHV-6 (50.9%), RSV (37.4%), EBV (35.5%), HBoV (28.2%), HHV-7 (18.3%) and rhinovirus (17.2%) were the most commonly detected viruses. Of 81 non- CAP samples tested, CMV (63%), RSV (49.4%), HHV-6 (42%), EBV (24.7%), HHV-7 (13.6%) and HBoV (8.6%) were the dominant viruses detected. The prevalence of several viral agents (rhinovirus, bocavirus, adenovirus and CMV) among IS samples of CAP were significantly higher than that of non-CAP control group. We also found the prevalence of RSV coinfection with HHVs was also higher among CAP group than that of non-CAP control.

**Conclusions:**

With sensitive molecular detection techniques and IS samples, high rates of viral identification were achieved in infants and young children with respiratory illness in a rural area of China. The clinical significance of rhinovirus, bocavirus, adenovirus and HHV (especially CMV) infections should receive greater attention in future treatment and prevention studies of CAP in infants and children.

## Introduction

Lower respiratory tract infections (primarily pneumonia) are the leading cause of death worldwide in infants and children [1], [2]. There are approximately 150 million cases of childhood community-acquired pneumonia (CAP) each year [1], [3]. CAP is a major cause of morbidity and mortality among children in developing countries, which is 10–50 times more common than in developed countries [1], [3]. Bacteria as the principal cause of CAP in children has been widely investigated [3]–[5]. In more than 50% of cases, however, there is still a considerable deficit in the aetiologic diagnosis resulting in unnecessary or inappropriate antibiotic prescription [2], [6]. It is clear that the involvement of viruses in CAP have been underestimated due to a lack of understanding of the viral etiology in a clinical setting [7]–[12]. In addition, the appropriate sample from infants and young children is critical for the aetiologic diagnosis of CAP. Lung itself is rarely sampled directly, and sputum, representing lower-airway secretions, can rarely be obtained from children [11], [14], [15].

Among children, CAP may be caused by a wide variety of microbes, including “typical” bacteria (e.g., Streptococcus pneumonia) and atypical bacteria, Mycobacterium tuberculosis and fungi. Viral infections are also involved with 80% of episodes of CAP in children under 2 years old and over 40% of older children [6]–[11]. Studies of CAP have traditionally focused little on viral causes [2]. So for, very few studies have included an extensive and appropriate evaluation of the role of viruses in the aetiology of CAP in developing countries, including China. In recent years, the introduction of better-quality diagnostic tests has markedly improved the ability to detect multiple viral pathogens [11]–[13], shifting attention to the important role of viruses as a cause of CAP [6]–[11]. According to previous studies, up to two-thirds of childhood pneumonia cases are associated with a viral infection [3]–[11]. Respiratory syncytial virus (RSV), influenza virus (IFV), rhinovirus (RV), human metapneumovirus (HMPV) and parainfluenza viruses (PIVs) are the most common viruses associated with pneumonia [3]–[20]. In addition, the roles of cytomegalovirus (CMV), other herpes viruses (HHVs), recently identified human coronaviruses (HCoV-NL63 and -HKU1) and human bocavirus (HBoV) as causes of CAP in infants and children remain controversial [3], [19]–[25]. So far, pathogenic profiles of HHV and its role in CAP among infants and young children from rural areas have not been well characterized. The present study was undertaken to describe the profiles of HHVs and other respiratory viruses associated with hospital-based CAP and non-CAP among infants and young children in a rural area of China using comprehensive and sensitive molecular diagnostic techniques.

## Materials and Methods

### Ethics Statement

All aspects of this study were performed in accordance with national ethics regulations and approved by the Institutional Review Boards of the Centre for Disease Control and Prevention of China and the Ethics Committee of Wenzhou Medical College. The participants received written information regarding the purpose of the study and of their right to confidentiality. Individual written informed consent was obtained from the parents or guardians of all participants.

### Subjects and Location

Wenling is located in a rural area on the southeast coast of China with a sub tropical monsoon climate. It has a population of approximately 1,000,000. According to World Health Organisation clinical criteria [3], [8], [11], CAP was defined as the presence of pneumonic infiltrates (alveolar or parenchymal) on chest radiography with simultaneous signs and/or symptoms of acute infection in which the reading of X-ray films by specialist were blinded to the clinical results. All CAP patients were also selected according to a set of necessary criteria based on respiratory symptoms (i.e., dyspnea or respiratory distress, cough, tachypnea) or evidence of parenchymal infiltrates on chest radiography. A total of 354 high-quality induced sputum (IS) samples (<25 squamous epithelial cells and >25 leukocytes per low-power field) were obtained from 948 hospitalised infants and young children with respiratory illness in Wenling Hospital from September of 2007 to April of 2008. Two hundred and seventy-three samples were preselected from hospitalised children patients who diagnosed as non typical bacterial CAP within 48 hrs of admission, while 81 samples from hospitalized children patients were set as a control group, whom were clinical diagnosis as non-CAP patients based on chest X-ray and other respiratory signs (asthma, chronic bronchitis or cystic fibrosis) at admission. Typical bacterial CAP based on microbiologic tests, treatment algorithms and an elevated leukocyte count (≥10^10^/L) were excluded. In addition, all patients were selected as immunocompetent at baseline and negative for HIV-1 and TB test. All the immunosuppressed or typical bacterial CAP patients were excluded. The children with presumed nosocomical CAP and lower-quality induced sputum (IS) samples were also excluded.

Sputum production was induced by the inhalation of a 5.0% hypertonic saline solution; the sputum was sampled during the 1^st^ week after hospital admission by aspiration through the nostrils. Our sputum collection method was described in detail elsewhere [14]–[16].

### Laboratory Methods

Nucleic acid was extracted from 200 µL of the virus transport medium (VTM) using a QIAamp MinElute Virus Spin Kit (Qiagen, Germany) according to the manufacturer’s instructions. Polymerase chain reaction (PCR) or multiplex PCR was performed as described previously [26]–[30] for HHVs, including HSV-1 and -2, varicella zoster virus (VZV), CMV, Epstein Barr virus (EBV), HHV-6 and -7. Adenoviruses (ADVs), IFV types A and B, PIV types 1–3, RSV, Picornaviruses (PIC, including enteroviruses and rhinoviruses) using multiple RT-PCR assays [26], [27] (Table S1); And for human coronavirus (HCoV)-OC43, -229E, -NL63 and -HKU1, human metapneumovirus (hMPV) using RT-PCR or and HBoV using nested-PCR assays [27]–[29] (Table S1). A positive (virus stock or DNA) and negative control (VTM only) in each set PCR assay was included to survey the possibility of laboratory contamination. All the methods were reported previously and validation in our lab [26]–[30].

The amplicons of positive for PIC were gel-purified for DNA sequencing using a QIAquick Gel Extraction (Qiagen, Germany), according to the manufacturer’s instructions. DNA sequencing was performed with specific primers using an ABI PRISM BigDye Terminator Cycle Sequencing Reaction kit (version 3.1) on an ABI PRISM 3130 DNA sequencer (Applied Biosystems, Foster City, CA), following the manufacturer’s instructions. Enteroviruses (EV) or rhinoviruses were identified based on sequence alignment of amplicons.

### Statistical Analysis

Eligibility and classification of the clinical syndromes of pneumonia were determined from the original record of each item on the medical history and examination in the database. The frequency distribution of viral pathogens between CAP and non-CAP were analysed by the χ^2^ test and Fisher exact test. All statistical analyses were performed with the Statistical Package for the Social Sciences (SPSS, Version17, SPSS Inc., Chicago, IL). Statistical significance was assessed by Tukey’s test and P-values <0.05 were considered to be statistically significant.

## Results

### Characteristics of the Study Population

All IS samples were collected from hospitalised patients with severe CAP (n = 273) and non-CAP (n = 81) in Wenling area between September 2007 and April 2008. The age and sex distributions are shown in Table 1. The mean age of the 273 CAP patients was 1.15 years (standard deviation, ±1.15 years, and 14 days to 11 years). The mean age of the 81 non- CAP patients was 3.31 years (standard deviation, ±2.91 years, 29 days to 13 years). Most CAP cases are infants with less than 12 months of age (186,68.13%) and male (180,64.9%). The mean admission day after fever was 8.05 days (standard deviation, ±9.53 days, and 1–61 days). About 52.7% of CAP patients received antibiotics before admission. None was positive for HIV-1 or TB.

**Table 1:** Demographic and aetiological data in this study.

Parameter		CAP		Non-CAP		P Value	
		No.	%	No.	%		
No. of cases		273		81			
Male/Female		180/93	64.9/34.1	49/32	60.5/39.5		
Mean age (Y)±SD (range)	1.15±1.51(14d-11y)			3.31±2.91(29d-13y)			
Age	≦6 m	122	44.69	10	12.3		
	6m–12 m	64	23.44	9	11.11		
	13–36 m	68	24.91	31	38.27		
	>36 m	19	6.96	31	38.27		
Any virus detected		272	99.6	80	98.76	0.406**	
Paramyxovirus	RSV	102	37.4	40	49.4	0.053*	
	PIV-1,2,3	8	2.9	0	0	0.206**	
	HMPV	17	6.2	4	4.9	0.794**	
Rhinovirus		47	17.2	3	3.7	**0.001** *	
Bocavirus		77	28.2	7	8.6	**0.001** *	
Influenza A		4	1.46	3	3.7	0.199**	
Influenza B		0	0	5	6.2	**0.001** **	
Herpesvirus	HSV(1/2)	7	2.9	0	0	0.359**	
	VZV	0	0	0	0	ND	
	EBV	97	35.5	20	24.7	0.069*	
	CMV	218	91.6	51	63	**0.002** *	
	HHV-6	139	50.9	34	42	0.157*	
	HHV-7	50	18.3	11	13.6	0.322*	
Adenovirus		19	7.0	0	0	**0.010** **	
Coronavirus	HCoV-229E	0	0	1	1.2	ND	
	HCoV-OC43	1	0.4	0	0	ND	
	HCoV-HKU1	0	0	0	0	ND	
	HCoV-NL63	0	0	1	1.2	ND	
Entervirus		3	1.1	0	0	ND	
Co-infection detected		241	88.28	65	80.25	0.064*	
	RSV+HHV	102/102	100	30/40	75	**0.001** **	
	RSV+HBoV	23/102	22.54	3/40	7.5	**0.037** *	
	RSV+Rhinovirus	4/102	0.39	0	0	**0.577** **	

* Pearson Chi-Square analysis using IBM SPSS Statistics 17.

** Fisher’s Exact Test analysis using IBM SPSS Statistics 17.

### Detection of Infection with HHVs and 15 other Respiratory Viruses

PCR substantially broadened the viral detection rate. With sputum samples as diagnostic specimens, viruses were identified in 272 (99.6%) of 273 CAP subjects while in 80 (98.76%) of 81 non-CAP control subjects as shown in Table 1. Of 273 CAP IS samples tested, CMV (250,91.6%), HHV-6 (139,50.9%), RSV (102,37.4%), EBV (97,35.5%), HBoV (77,28.2%), HHV-7 (50,18.3%) and RV (47,17.2%) were the most commonly detected viruses. ADV (19,7%), hMPV (17,6.2%), HPIV (8,2.9%), HSV-1/2 (8,2.9%), INF A/B (7,2.6%), EV (3,1.1%) and HCoV-OC43 (1,0.4%) were also found. No samples were positive for HCoV-229E, -NL63, -HKU1 or VZV. Of 81 tested non-CAP IS samples, CMV (51,63%), RSV (40,49.4%), HHV-6 (34,42%), EBV (20,24.7%), and HHV-7 (11,13.6%) were dominant viruses. INF (8,9.9%; INF A/B, 3/5), HBoV (7,8.6%), hMPV (4,4.9%), rhinovirus (3,3.7%), HCov-229E (1,1.2%) and HCoV-NL63 (1,1.2%) were also detected. No non-CAP IS samples were positive for HPIV, HSV-1/2,VZV, EV, ADV, HCoV-OC43 and HCoV-HKU1.In addition, we found that the prevalence of several viral agents (rhinovirus, HBoV, ADV and CMV) among IS samples of CAP were significantly higher than that of non-CAP control group (*P*<0.05), while the prevalence of INF B (5,6.2%) among IS samples of non-CAP were significantly higher than that of CAP group (*P* = 0.001).

A total of 271 CAP cases were positive for HHVs, accounting for about 99.3% of the hospitalised infants and young children with CAP included in this study. 73 of 81 non-CAP cases, however, were also identified as positive (90.1%) for HHVs. Among other 15 respiratory viruses, RSV was the most dominant for both CAP and non-CAP groups, which was present significantly less frequent than HHVs.

### Distribution of Viruses by Age and Season

**Figure 1:**
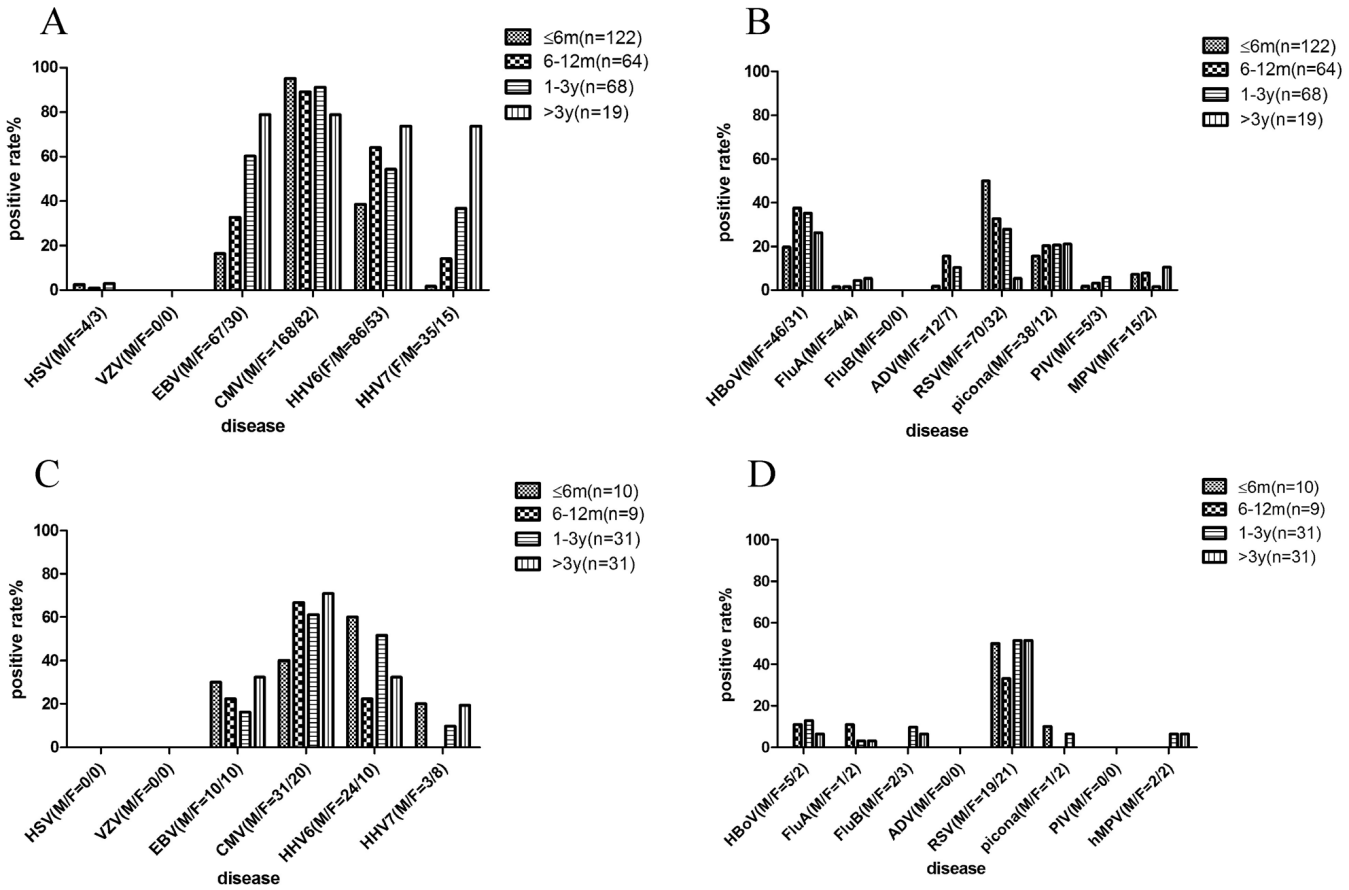
Distribution of viruses among different age groups in this study. (A) Detection HHVs in CAP group; (B) Detection common respiratory viruses in CAP group; (C) Detection of HHVs in non-CAP group; (D) Detection common respiratory viruses in non-CAP group.

**Figure 2:**
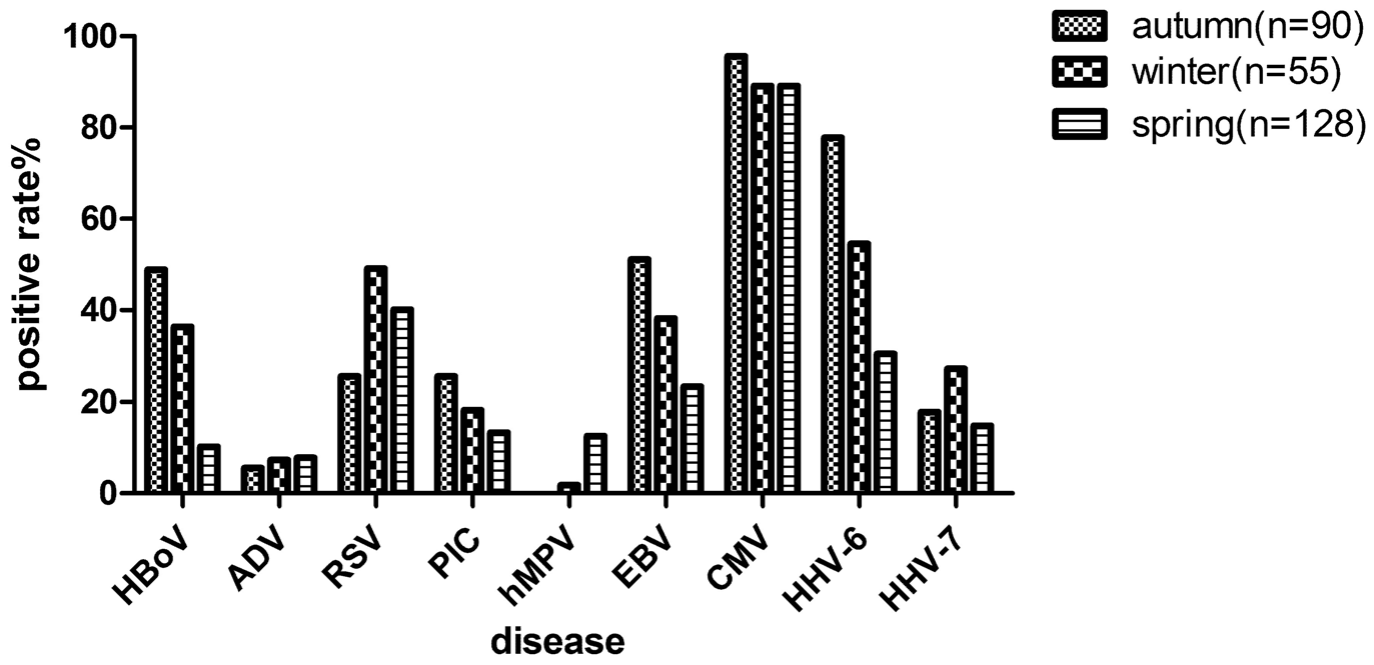
Seasons distribution of dominant viruses in IS samples of CAP patients from September of 2007 to April of 2008(Autumn: Sep. to Oct. of 2007; Winter: Nov. of 2007 to Jan. of 2008; Spring: Feb. to Apr. of 2008).

To further study the epidemiological profiles of virus infections, the distribution of viruses by age and season in this study were characterised (Figure 1 and 2).

No significant difference was found for FluA, Piconavirus (enterovirus/rhinovirus), PIV and hMPV among various age groups of CAP cases (Figure 1A). However, the infection rate of HBoV and ADV showed a peak among CAP patients aged 6 months to 3 years (P<0.05). In contrast, RSV detection peaked in the infant group (0–12 months) of CAP and decreased significantly with advancing age. In addition, no significant differences for HSV and CMV were observed among various age groups of CAP cases (Figure 1B). However, the rate of infection with EBV, HHV-6 and HHV-7 increased with age among CAP cases (P<0.05). The infection rate was more than 73.7% for CMV, EBV, HHV-6 and HHV-7 among children older than 3 years.

The distribution of viruses by age among the control group with non-CAP was also investigated (Figure 1C and 1D). No significant differences for RSV, EBV, HHV-6 and CMV were shown among various age groups of non-CAP cases. However, the rate of infection with HHV-7 increased with age, which is similar to that of CAP group.

Among IS samples of CAP cases, the seasonal distribution of viruses was investigated (Figure 2). From September of 2007 to April of 2008, it was found that the peaks of RSV in winter of 2007 and spring of 2008, HBoV and PIC in autumn of 2007, and HMPV in spring of 2008. In addition, EBV and HHV-6 were more frequently detected in autumn of 2007. HHV-7 infection reached its height during winter of 2007. However, we found no significant difference in CMV detection among samples from different seasons.

### Co-infection and Clinical Profiles

Interestingly, co-infections were found in 241 (88.28%) of the CAP cases and 65 (80.25%) of the non-CAP cases (Table 1). Single virus infection were detected in 31 IS samples of CAP cases (25 for CMV only, 5 for HHV-6 only and 1 for HBoV only). In this study of co-infection, 77 patients were found to be infected with 2 viruses, 76 with 3 viruses, 48 with 4 viruses, 36 with 5 viruses, 3 with 6 viruses and 1 with 7 viruses. In addition, HHVs were the most detected co-infection agent with other respiratory viruses. Among 102 RSV infections of CAP cases, HHV (102 cases, 100%) and HBoV (23 cases, 22.54%) were the most common concomitantly detected viruses, which were significantly higher than that of coinfection among non-CAP group.

The clinical manifestation of CAP patients included cough, fever (≥38°C), asthma and sputum. A few cases also showed signs of diarrhoea, rhinorrhoea, dyspnea and rale (data not shown). Since the high virus detection rate (more than 98%) and co-infection rate (more than 80%) were among IS samples from both CAP and non-CAP groups in this study (Table 1), it was difficult to associate the clinical symptoms of patients with CAP with individual virus infection. However, the prevalence of several viral agents (rhinovirus, HBoV, ADV and CMV) among IS samples of CAP were drastically higher that of non-CAP control group (P<0.05). In addition, the prevalence of RSV coinfection with HHVs (102/102, 100%) and HBoV (23/102,22.54%) among CAP group was significantly higher than that among non-CAP controls (P<0.05).These data indicated that several viral agents (such as HBoV and CMV) may contribute to the occurrence of CAP.

## Discussion

CAP is more common and severe in the developing areas than developed areas [1]–[3], and is a major cause of death among infants and children in rural areas [3]. Previous investigations of paediatric CAP in US and Europe emphasised the importance of infections with common respiratory viruses (RSV, INF, PIV and ADV) [3], [4], [6]. The roles of HHVs and more recently identified viruses (HBoV, HCoV-NL63 and HCoV-HKU1) as causes of CAP remain controversial [2], [5]–[7], [12], [14]. In this study, the viral prevalence in sputum specimens of childhood with non typical bacterial CAP was investigated using sensitive molecular diagnostic methods for HHVs and 15 respiratory viruses, and viruses were detected in 99.6% of the children. This is not surprising considering that the samples included were highly selected for the discovery of viral etiology and addition HHVs detection in this study. To our knowledge, this is the first comprehensive study of the prevalence of HHVs in sputum samples among infants and young children with CAP [2]–[15].

A few reports have described the detection of DNA from several HHVs in respiratory samples, with most of them focusing on immunosuppressed individuals or adults with CAP [2], [19]–[22], [31], [32]. In this study, we screened for DNA of HHVs in IS samples among infants and young children with CAP using a sensitive multiple PCR assay; of the HHVs considered, only VZV was not detected. The highest positive rate was found for CMV infection (91.6%). CMV infection, which is usually congenital, showed no significant difference among various age groups in this study. These data are consistent with those of previous reports [2], [22], [31]. In addition, it was reported here that CMV and HHV-6 were the only detected viral agent among 25 CAP cases and 5 CAP cases, respectively. Furthermore, the prevalence of CMV among IS samples of CAP were significantly higher than that of non-CAP control group. The prevalence of RSV coinfection with HHVs among CAP group was drastically higher than that among non-CAP control (P<0.05). These data suggest an association between infection with HHVs (especially CMV) and CAP in infants and children.

Few comprehensive studies have searched for viruses in IS samples among infants and children with CAP in rural areas [8], [10], [11]. In this study, 15 common and recently identified viruses associated with acute respiratory infection were screened using molecular methods. Our results are consistent with previous studies conducted in China or other areas [8], [10], [11], [33], which showed that the most-detected agent was RSV, followed by HBoV, RV, HMPV, ADV and PIV3 in IS samples from infants and children with CAP [3], [11], [14]–[16]. RSV and HBoV were also the dominant viruses detected in IS samples from non-CAP group. Moreover, our data show a higher HBoV detection rate (28.2%) compared with previous reports [23], [24]. The prevalence of HBoV among IS samples of CAP were significantly higher that of non-CAP control group. Similar trends were also observed in the prevalence of rhinovirus and ADV among IS samples of CAP when compared with non-CAP control group. Unlike previous data [34], the prevalence of HCoVs among IS samples was significantly lower in present study. These differences might primarily due to the specimens [11], [15], [16]–IS vs. a nasopharyngeal aspirate or nasopharyngeal wash. In the meantime, the impact of other factors, such as area and the duration of the study period on infection, could not be ruled out.

One interesting finding of present study was that HHV co-infections were found in 70% of the CAP cases, compared with rates between 15 and 45% in previous etiological studies of childhood CAP [7], [35]–[37]. The clinical consequences of mixed infections have not been fully understood yet. Evidence suggests that mixed viral infections can lead to more severe condition than individual viral infections [2], [7], [35]–[38]. In this study, it was unable to determine whether the HHVs were reactivated from a latent reservoir after another respiratory virus infection, or if an immunosuppressed condition caused by HHV infection increases the potential risk of other respiratory virus infections. Consequently, it is difficult to estimate the true association between the clinical manifestations and virus infection. At the same time, we understand that the detection of viruses in an IS sample by PCR does not necessarily mean that they are the causative agents of the concomitant CAP.

The IS samples included in this study may only represent part of hospitalized infants and children with CAP in this hospital. To evaluate the real pathogenic role played by virus in hospitalized children with CAP in this study, non-CAP hospitalized children with chronic respiratory illness were set as control group. However, this study still has two major limitations. One limitation of this study is that viral detection from non-hospitalised children (due to limitation of ethics) and patients with bacterial CAP, which would have provided a control group for this study, were not included. Therefore, some viral agents detected in this study may represent asymptomatic persistence, prolonged shedding, or other situations. It is also not possible to evaluate the exact importance of each virus responsible for CAP in most cases. Nevertheless, it is believed that overall this study highlights the importance of HHVs (mainly CMV) and respiratory viruses in Children with CAP. Another limitation of this study is that no samples were collected during summer, which could lead to the missing of some viral agents such as parainfluenza viruses and some enteroviruses.

## Conclusions

In summary, our study on the prevalence of HHVs and other respiratory viruses in infants and young children with CAP identified a detectable virus in more than 99.6% of case participants, in which CMV, HHV-6, EBV, RSV and HBoV were clearly predominant (>25%) and contributed significantly to the spectrum of CAP in a rural area of China. Although the HHVs were the most commonly identified pathogens in this study, which were not previously thought of as typical causes of CAP in immunocopetent individuals [3]–[8], [20],[38]–[41], further studies are required to determine the relationship of the presence of HHVs and severity of disease, thus the clinical significance of HHV infections or co-infections should receive greater attention in future treatment and prevention studies of CAP in infants and children.

## Supporting Information

Table S1 Primers (5′-3′) and Targets Used for the Detection of Respiratory Viruses in the Study.
(DOCX)
