# Supplementary material for: Prevalence of Herpes and Respiratory Viruses in Induced Sputum among Hospitalized Children with Non Typical Bacterial Community-Acquired Pneumonia
Source: PLoS One. 2013 Nov 18;8(11):e79477. doi: 10.1371/journal.pone.0079477 (PMC3832587; doi:10.1371/journal.pone.0079477)
Supplement: Table S1 — Primers (5′-3′) and Targets Used for the Detection of Respiratory Viruses in the Study. (DOCX) [file pone.0079477.s001.docx]

**Supplementary data**

**Table S1. Primers (5’-3’)and Targets Used for the Detection of Respiratory Viruses in the Study.**

| **Assays and** |  | **Primer* and probe** | **Target genes** | **Ref** |
| --- | --- | --- | --- | --- |
| **Viruses detected** | |  |  |  |
| **RT-PCR** |  |  |  | 27 |
| **HCoV-229E** | 229E-F | TAGGTTTTGACAAGCCTCAGGAAAAAGA | N |  |
|  | 229E3-R | GTGACTATCAAACAGCATAGCAGCTGT |  |  |
| **HCoV-OC43** | OC43E-F | GTAAGAGAGGCCCTAATCAGAA | N |  |
|  | OC43-R | CTTCATTCATTTACTAATTACTGG |  |  |
| **HCoV-HKU1** | HKU1-F | TAGTGGTATGGATACTGCCTTGT | POL |  |
|  | HKU1-R | ACAAGGCAGTATCCATACCACTA |  |  |
| **HCoV-NL63** | NL63-F | CTGTTACTTTGGCTTTAAAGAACTTAGG | N |  |
|  | NL63-R | CTCACTATCAAAGAATAACGCAGCCTG |  |  |
| **Multiple- PCR** |  |  | DNA POL | 26 |
| **HSV1,2** | HSV-F | GCCAAGAAAAAGTACATCGGCGTCATC |  |  |
|  | HSV-R | TGAGGACAAAGTCCTGGATGTCCCTCT |  |  |
| **VZV** | VZV-F | TCCGACATGCAGTCAATTTCAACGTC |  |  |
|  | VZV-R | GGTCGGGTAGACGCTACCACTCGTTT |  |  |
| **EBV** | EBV-F | CTTAGAATGGTGGCCGGGCTGTAAAAT |  |  |
|  | EBV-R | ATCCAGTACGTCTTTGTGGAGCCCAAG |  |  |
| **CMV** | CMV-F | GCGCGTACCGTTGAAAGAAAAGCATAA |  |  |
|  | CMV-R | TGGGCACTCGGGTCTTCATCTCTTYAC |  |  |
| **HHV6** | HHV6-F | ATGCGCCATCATAATGCTCGGATACA |  |  |
|  | HHV6-R | CCCTGCATTCTTACGGAAGCAAAACG |  |  |
| **HHV7** | HHV7-F | GCCCGTTTTCGGAAATATTGGAGAGAT |  |  |
|  | HHV7-R | ACGCACGAGACGCACTTTTCTTAAACA |  |  |
| **Multiple-nested PCR** | |  |  |  |
| **Mix1 FluA** | FA-1F | CAGAGACTTGARRATGTYTTTGC | Matrix | 27 |
|  | FA-1R | GGCAAGYGCACCRGYWGARTARCT |  |  |
|  | FA-2F | GACCRATCCTGTCACCTCTGACT |  |  |
|  | FA-2R | AYYTCYTT GC CCATGGAATGT |  |  |
| **FluB** | FB-1F | GTGACTGGTGTGATACCACT | HA |  |
|  | FB-1R | TGTTTTCACCCATATTGGGC |  |  |
|  | FB-2F | CATTTTGCAAATCTCAAAGG |  |  |
|  | FB-2R | TGGAGGCAATCTGCTTCACC |  |  |
| **ADV** | AD-1F | GCCGCAGTGGTCTTACATGCACATC | Hexon |  |
|  | AD-1R | CAGCACGCCGCGGATGTCAAAGT |  |  |
|  | AD-2F | GCCACCGAGACGTACTTCAGCCTG |  |  |
|  | AD-2R | TTGTACGAGTACGCGGTATCCTCGCGGTC |  |  |
|  | AD-2F9 | CMGASACSTACTTCAGYMTG |  |  |
|  | AD-2R9 | GTASGYRKTRTCYTCSCGGTC |  |  |
| **Mix2 hRSV** | RS-1F | TGGGAGARGTRGCTCCAGAATACAGGC | N | 27 |
|  | RS-1R | ARCATYACTTGCCCTGMACCATAGGC |  |  |
|  | RS-2F | ACYAAATTAGCAGCAGGG |  |  |
|  | RS-2R | CTCTKGTWGAWGATTGTGC |  |  |
| **PIC** | PIC-1F | GCACTTCTGTTTCCCC | 5’-UTR |  |
|  | PIC-1R | CGGACACCCAAAGTAG |  |  |
|  | PIC-2F | GCACTTCTGTTTCCCC |  |  |
| **Mix3 PIV-(1-3）** | P123-1F | GTWCAAGGAGAYAATCARGC | L | 27 |
|  | P123-1R | GRTCYGGAGTTTCWARWCC |  |  |
| **PIV-1** | P1-2F | GCATCAGACCCTTATTCATG |  |  |
|  | P1-2R | GTTGTATCAAGCATCCCGGC |  |  |
| **PIV-2** | P2-2F | CAGCCGATCCATACTCATTG |  |  |
|  | P2-2R | CTTGTGGTGTCAAAAAATCC |  |  |
| **PIV-3** | P3-2F | GCTGTTACTACAAGAGTACC |  |  |
|  | P3-2R | GTTGCCAGATTTGAGGATGC |  |  |
| **RT-PCR** |  |  |  |  |
| **hMPV** | hMPV-F | AACCGTGTACTAAGTGATGCACTC | N | 28 |
|  | hMPV-R | CATTGTTTGACCGGCCCCATAA |  |  |
| **Nested- PCR** |  |  |  |  |
| **HBoV** | HBoV-1F | CCAGCAAGTCCTCCAAACTCACCTGC | NP-1 | 29 |
|  | HBoV-1R | GGAGCTTCAGGATTGGAAGCTCTGTG |  |  |
|  | HBoV-2F | GACCTCTGTAAGTACTATTAC |  |  |
|  | HBoV-2R | CTCTGTGTTGACTGAATACAG |  |  |

*K = G+T, M = A+C, R = A+G, S = G+C, W = A+T, Y = C+T.

1st round primers:-1F,-1R; 2 nd round primers: -2F or -2F9, -2R or -2R9.
